# Gaps in asthma diagnosis and treatment in low- and middle-income countries

**DOI:** 10.3389/falgy.2023.1240259

**Published:** 2023-10-23

**Authors:** Monica Barne

**Affiliations:** Department of Training, Pulmocare Research and Education Foundation, Pune, India

**Keywords:** asthma, gaps, low- and middle-income countries, factors, challenges

## Abstract

Low- and middle-income countries (LMICs) contribute to a major proportion of asthma morbidity and mortality globally, even though the prevalence is higher in high income countries. Mortality due to asthma is avoidable and patients should be able to live a near normal life. There are factors that influence overall disease prevalence and poor health outcomes due to asthma in LMICs. This article summarizes the gaps in asthma diagnosis and management in LMICs. The gaps are diverse. Each challenge needs to be addressed through policy decisions, upgrade of infrastructure, knowledge and skills for early diagnosis and correct management among health care providers, both clinicians and paramedics. Healthcare accessibility and affordability are genuine challenges, and the public healthcare system needs to be geared up to address these at primary and tertiary levels. Mass education of the population through national level government initiatives is needed to help bridge the sociocultural gaps.

## Introduction

Globally, asthma is the most prevalent chronic respiratory disease (1). 262.4 million people suffer from asthma worldwide (1). The recent Global Asthma Network (GAN) Phase-1 study (2) reports a higher prevalence of asthma in high income countries as compared to low-and middle-income countries (LMICs). However, severe asthma and high mortality due to asthma is seen more in LMICs (1, 2). 96% of global asthma mortality occurs in LMICs (3). This avers that in LMICs, there are likely to be several gaps in diagnosis and management of asthma which may be leading to poorly controlled asthma, severe asthma, poor quality of life and asthma mortality, which is avoidable, (4) as is the economic burden, if asthma is managed appropriately (5).

**Figure 1:**
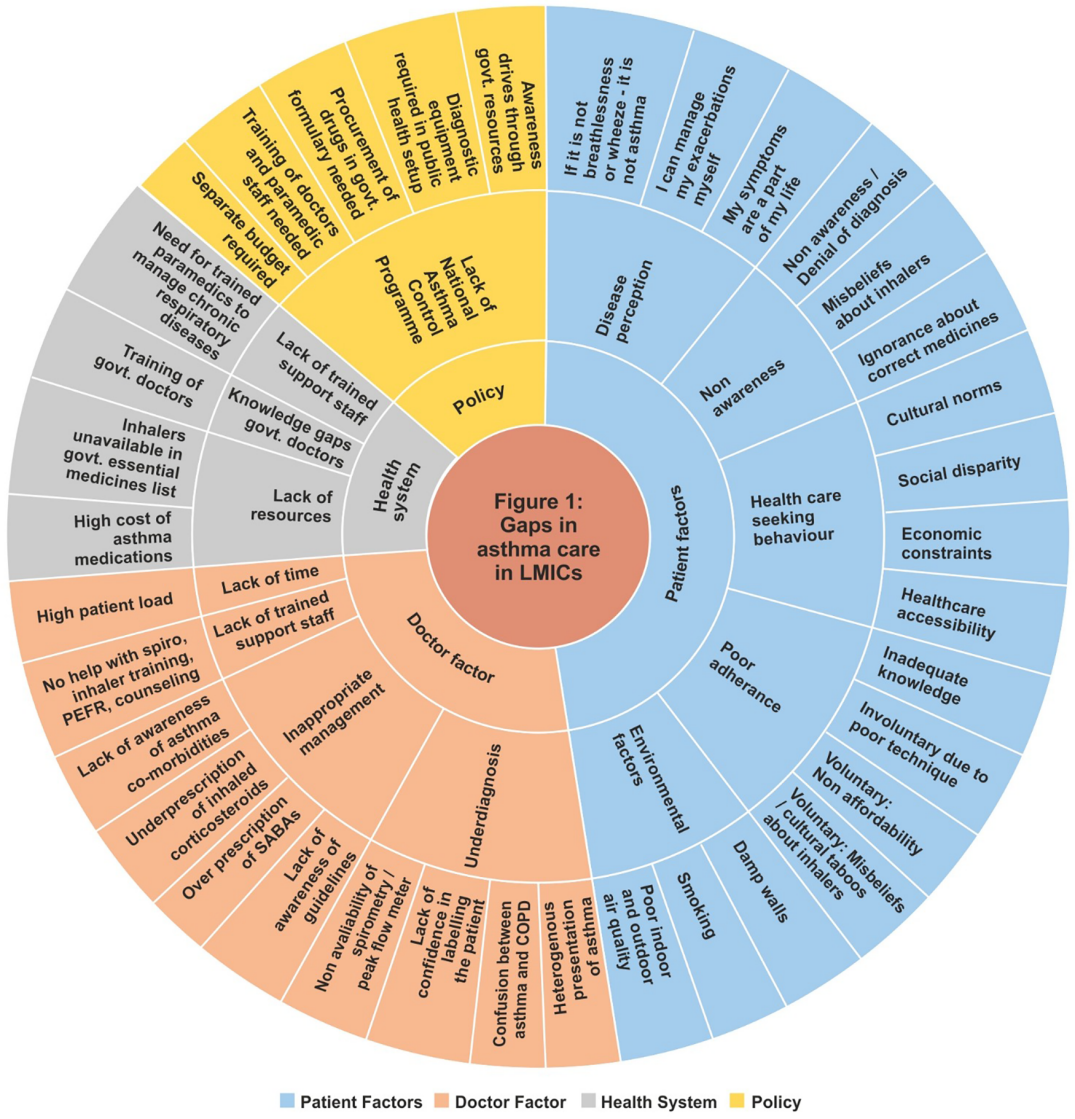
Gaps in asthma care in LMICs—a bird's eye view.

The gaps are multifaceted, multifactorial and can be classified as patient related, healthcare provider related, healthcare systems related, and policy related. Figure 1 gives a bird's eye view of the gaps.

## Patient factors

Asthma is associated with several stigmata due to which, patients are not willing to accept the diagnosis (6). Patients and their care givers believe asthma to be a debilitating condition, incurable, often fatal and they prefer not to have a diagnosis of asthma (7). In LMICs, societal and cultural norms are at odds for asthma particularly if it's a woman suffering (6). Patient perception of the condition is a major factor for poor control (8). Despite persistent symptoms, patients of asthma do not realize that they are uncontrolled (9, 10). Many ignore the warning signs of an acute exacerbation and believe they will be able to manage their exacerbations on their own (10). They lack awareness about what the correct treatment for asthma is. In the Asia Pacific Asthma Insights and Management (APAIM) Survey, in India, though 83% and 68% patients respectively reported to be taking reliever and controller medications, 89% were taking these orally. This may explain why India reported the highest number of exacerbations, loss of work or school and night-time awakenings, with the worst level of asthma control amongst the nine countries studied in the APAIM (11). In a study conducted in Nigeria, use of over-the-counter oral steroids as a quick fix for symptoms was the second strongest predictor of poor asthma control (12). Use of systemic corticosteroids (SCS) both short term and long term is associated with several side effects like cardiovascular events, osteoporosis, metabolic syndrome, diabetes etc. SCS have also been shown to increase dose-dependent all-cause mortality among asthmatics (13).

Health care seeking behavior of asthmatics in LMICs is poor. Most prefer to go to the government sector which, in most LMICs, is not well equipped to handle asthma. Private sector health facilities are better equipped but are accessed mainly by patients from the upper socio-economic strata (14, 15). Most patients remain unaware of their diagnosis until they reach a tertiary care center (14). Even in high-income countries, the South Asian population which largely comprise expatriates from LMICs has a poorer health seeking behavior. Most rely on care from family even in exacerbations or prefer alternative medicine (6, 16). At a government hospital in Zimbabwe, only 30% of all patients seeking asthma care came from the rural area due to challenges in accessibility. Several social, economic, and cultural aspects make it difficult for people in rural areas to access healthcare (17).

Poor adherence is a major factor causing poor control of the disease. Involuntary poor adherence occurs when patients do not use their inhalers correctly (18, 19) or have not understood the dosage regimen. Dhadge et al. from India found that despite appropriate training, patients made critical mistakes in use of inhalers within the first 24 h which sequentially increased at 7 days and 28 days respectively (20). Fear of side effects, non-awareness about the need to continue inhalers despite good symptom control, fear of addiction, are some reasons why patients voluntarily discontinue their asthma medications (21, 22). Non-availability of inhalers and economic constraints are responsible for poor adherence in LMICs (12, 23). Inhaled salbutamol and budesonide respectively cost between 0.5 and 1 day worth of daily wages lost which is difficult for daily wage workers to afford (24). Poor health literacy and unrealistic expectation of cure rather than control also leads to poor adherence (25).

Lifestyle related factors specific to LMICs cannot be ignored. In a study conducted in Puducherry (Pondicherry) in South- India, lack of proper exhaust in the kitchen was implicated in development of asthma (26). Biomass fuel was responsible for lack of asthma control and increased severity of asthma in Nigeria (27). Poor indoor air quality, higher prevalence of fungal sensitization, presence of damp walls, presence of molds, third hand smoking are all associated with development of, and poor control of asthma (28, 29). Maternal smoking was the only independent risk factor associated with hospital admissions due to asthma exacerbation in children in Columbia (30). In absence of a family history of atopy, lack of knowledge of these environmental factors that increase the risk of asthma, can lead to denial of the diagnosis (7).

## Physician related factors

Asthma suffers gross underdiagnosis and misdiagnosis. Only a fraction of patients with symptoms of asthma are ever diagnosed as asthma by their health care providers. In the GAN Phase 1 study conducted at nine centers in India, 82% of current wheezers and 70% of patients with severe asthma were not clinically diagnosed to have asthma (31). Arjun et al. have found that only 11.3% patients had a diagnosis written despite having received treatment for asthma (14). The reasons for this may be manyfold.

In LMICs, the doctor-patient ratio is low (32). Asthma is a chronic condition which requires unconventional form of therapy, for long term and suffers several myths and misconceptions amongst the population. To manage asthma, the barriers of these myths need to be broken and patient concordance should be obtained. Doctors need time for this, which they do not have due to a huge burden of patients. In most LMICs doctors spend just about 2–3 min with each patient. An average duration that a practitioner in Bangladesh gives a patient is just 48 s which is the lowest globally. Afghanistan, China, India, all fall short of even 5 min (33). Within this short time span, it is impossible for a primary healthcare provider to take a good clinical history, perform spirometry or peak flow reading, conclude the diagnosis, prescribe correctly, teach inhaler technique, and explain to the patient about the disease. An easier way which most resort to, is to continue to prescribe the conventional though inferior medications. If they refer their patients to a specialist, they risk losing them, which is detrimental to their practice. If the doctor labels the case as asthma or prescribes inhalers without proper counselling, patients are likely to go doctor shopping if they disagree with the doctor's diagnosis or do not believe in the treatment (34).

The heterogenous presentation of asthma is often confusing. Asthma is inappropriately perceived as a disease of breathlessness and wheeze, mandatorily associated with family history of allergy (7). In LMICs, pediatric asthma cases are often misdiagnosed as pneumonia or bronchiolitis (35). Physicians are unable to differentiate between asthma and Chronic Obstructive Pulmonary Disease (COPD) and often use the terms interchangeably. In a tertiary care hospital in South Africa, one out of every five patients was labelled as asthma during one visit and COPD during the next (36). This study also reported that those asthmatics who were labelled appropriately remained well controlled on regular controller therapy emphasizing the need for proper diagnosis and labelling.

Every patient with an acute exacerbation should be initiated on controller therapy. However, in LMICs, adherence to guideline-based management of asthma exacerbations is poor. In an audit conducted in a Nigerian hospital regarding management of exacerbations, only 19.1% patients were prescribed controller therapy at discharge while most (58.8%) were discharged on oral steroids only (37). In Ecuador, very few of the participants who had been enrolled in a study at their first exacerbation, had received controller therapy (38).

Spirometry, the objective diagnostic tool for asthma, is not yet optimally utilized by healthcare providers. Limited availability, lack of trained manpower to perform spirometry, poor understanding of interpretation of the test, perceived relatively high costs, all contribute to underutilization of spirometry (39, 40). The Global Initiative for Asthma (GINA) (41) recommends use of peak expiratory flow meter (PEFM) to demonstrate variable airflow obstruction for diagnosis of asthma in resource limited setting. However, PEFM is not yet being utilized due to lack of knowledge of its clinical utility and poor availability particularly in LMICs (42).

Even among those diagnosed with asthma, only a fraction receives guideline-based therapy. In a prescription audit conducted in India it was found that only 10%, 26% and 7% prescriptions from graduate, postgraduate and superspecialist physicians respectively, contained inhaled corticosteroids (ICS) + long-acting beta_2_ agonists (LABA) (43). In South Africa, only 38% of patients with mild asthma were prescribed ICS. Market analysis of prescribed medication for respiratory diseases showed dominance of prescription of short-acting beta_2_ agonists (SABAs) over ICS by 3.6 times (44). Salvi et al. in India found that only 6.8% of asthma patients were procuring ICS for their asthma treatment. Moreover, state wise analysis and comparison with mortality and disability adjusted life years (DALYs) showed that lower the sales of ICS, higher was the mortality and DALYs due to asthma in the state (45).

There is an overuse of SABAs and other bronchodilators in LMICs. 93% of asthma prescriptions audited in a tertiary care hospital in Gujarat, a western state in India, contained a prescription of oral methylxanthines and 28% had oral salbutamol. 71% prescriptions did have a combination of ICS and LABA, but most patients were also given unwarranted prescriptions of an average of 4.75 drugs consisting oral xanthines, oral steroids, antibiotics, antihistamines, H2 blockers or proton pump inhibitors without any history of concomitant gastroesophageal co-morbidity or infections or allergies (46). In a tertiary care hospital in Pune, India, SABAs were found to be over prescribed (≥3 canisters per year) in about one fourth of the patients (47). Similarly, in seven cities in Columbia, 43.2% patients used three or more than three canisters of SABA in a year and 25.2% were prescribed ≥10 canisters (48). Additionally, over-the-counter procurement of SABA was done by 8% and 17.6% in Pune and Columbia respectively. Similar overprescription of SABA was associated with poor health outcomes in Africa and Latin America (49, 50).

Emerging comorbidities and newer phenotypes may be complicating management for doctors. In the GAN Phase 1 study in India, prevalence of Allergic Rhinitis (AR) was about 9.8% amongst adults. Presence of AR was associated with a 4.5 times higher risk of having wheeze in the past 12 months and about 2.5 times higher need for nebulized medication for relief of their symptoms (51). In a study conducted among adolescent school children using a questionnaire and spirometry in the highly polluted city of Delhi in India, the only significant risk factor that was associated with development of asthma was high body mass index (BMI) (52). Global Burden of Diseases (GBD) data has shown that a high BMI increases the risk of asthma mortality and morbidity and is an emerging factor that doctors in LMICs may not be aware of (53).

All these factors warrant need for continuous medical education programmes for practicing clinicians. Evidence based, updated, interactive programmes either online or physical, have been shown to bridge the knowledge and practice gaps in asthma diagnosis and management. The pharmaceutical industry plays an important role in keeping clinicians updated with educational material about latest guidelines and information about newer and safer drugs that are being added to the armamentarium of asthma management. The industry may also support by disseminating this information through educational activities to ensure that doctors are updated with latest advances, and patients receive state of the art care (54–56).

Trained technicians or respiratory therapists can support physicians in performing spirometry, training patients on how to use peak flow meter and inhaler devices, checking device technique during follow ups and providing asthma education to patients. In India, however, trained respiratory therapists are usually engaged in acute care, mostly in intensive care units at private hospitals (57). Government hospitals do not have a designated post for a respiratory therapist or a pulmonary function technician. The role of asthma nurses in improving health outcomes in asthma patients from high-income countries is well established. A similar cadre needs to be created in LMICs.

In India, medical schools focus more on infectious diseases, mainly tuberculosis and pneumonias, even though most patients seen in respiratory outpatient departments are of asthma (29.8%) rather than COPD (15.6%) or respiratory tract infections (11.3%) or tuberculosis (8.7%) (15). GBD data has shown an increase in prevalence of asthma between 1990 and 2016 (58) while the prevalence of tuberculosis in India has decreased significantly in two decades from 1990 to 2019 (59). Yet respiratory departments are predominantly considered as departments of tuberculosis. For medical students, because rotations in respiratory department is not compulsory, students do not learn how to diagnose and manage asthma appropriately. They do not know the correct technique of using inhaler devices. In a study conducted in a tertiary care center in India, less than 2% of graduate and post graduate students of medicine knew how to use a pressurized metered dose inhaler (pMDI) or a pMDI + spacer correctly (60). In a tertiary care hospital in Mumbai, India, the healthcare providers including physicians fared just as bad as the patients in use of inhaler devices (61). There is a need to have focused, skill-based training sessions on diagnosis and management of asthma for medical students.

## Health systems

There is an unequitable distribution of resources between government and private sectors in LMICs. The gold standard diagnostic test, spirometry, is not available in rural areas. Even in urban areas, spirometry is available in limited tertiary care centers which usually lack skilled technicians who can perform acceptable spirometry tests (62). There is disparity in availability of inhalers in the public health care system which is the primary healthcare provider system for rural and economically challenged patients (23, 63–65). In many LMICs, basic asthma medications are absent in the essential medications list (66). A systematic review of data from 60 LMICs found that asthma medications were either not available or were too costly. Salbutamol inhaler costed around 1–4 days' wages, ICS cost about 2–7 days' wages, and a combination inhaler of ICS + LABA cost at least 6 days' of daily wages which is largely unaffordable for patients of low socioeconomic strata (67). There are differences in the resource distribution in urban and rural areas (14, 26, 68, 69). Even in high income countries, low economic status is known to have a poor health outcome with higher exacerbations rates and more emergency visits (70). Sri Lanka, however, is an exception and has achieved equitable and affordable distribution of essential drugs for asthma in both public and private sector indicating optimistic possibilities for public health facilities of other LMICs (71).

## Health policies

Government health departments in LMICs have a larger focus on communicable diseases. In India, the first government programme for non-communicable diseases called as National Programme for Prevention & Control of Cancer, Diabetes, Cardiovascular Diseases & Stroke (NPCDCS) was launched in 2010 (72). Chronic respiratory diseases, particularly COPD and asthma have been included in 2016 and the detailed guidelines have been issued as recently as 2021 (73). However, asthma is but a component of the NPCDCS though it deserves a separate National Asthma Control Programme. Such a program could help address the challenges and gaps in asthma management using a multi-pronged approach like the Asthma Right Care movement initiated by the International Primary Care Respiratory Group. Asthma right care is a multidisciplinary approach to improve asthma healthcare by first drawing attention to all the lacunae and challenges, and then initiating a dialogue between all stake holders including patients, healthcare providers, pharmacists, researchers, and policy makers to address those (74). The Programme for Control of Asthma in Bahia (ProAR) is another such example of a public health initiative supported by the Government which intervened at multiple levels in the city of Bahia in Salvador province of Brazil with free medical care, pharmaceutical assistance (inhaled medication), patient education, healthcare skill development and research to significantly reduce the asthma hospitalizations by 82.3% within a span of 8 years (75). The World Allergy Organization has raised a call to action to reduce the use of systemic corticosteroids through optimization of ICS, using SCS sparing strategies like addition of LABAs and biologics wherever applicable (13). A paradigm of successful health systems intervention is of The Finnish Asthma Programme initiated in 1994. Through upgrading knowledge of health care providers for early detection and correct treatment using ICS, education of patients, public awareness campaigns and environmental control The Finnish Asthma programme reduced the burden of disease over two decades, with a reduced duration of hospitalizations by 54% and reduced yearly cost per patient by 72% despite an increase in prevalence of asthma (76).

## Conclusion

Asthma is a common respiratory condition unique due to its varied presentation, challenging diagnosis, unconventional form of therapy, long term nature and at best control but no cure. Additional challenges in LMICs are social stigma, lack of awareness amongst the population and health care providers alike, unequitable distribution of resources and perceived low priority amongst policy makers and government. It is time for interventions at all levels through a National Asthma Control Programme. Under the aegis of this programme, health ministries could initiate measures for upgrading infrastructure and upskilling resources for proper diagnosis and management of asthma. Diagnostic equipment, essential inhaled drugs and devices could be made available at the public health centers. Mass media campaigns for awareness can be driven. In the private sector, upgrading knowledge and practice skills of primary care doctors through continuous medical education programmes in public private partnerships can be initiated. Policy makers and medical societies can develop simple and practical diagnostic and management protocols and encourage implementation in public and private primary care sectors to ensure a standard level of care. Focus on asthma should be increased at medical school level and all students should be taught the correct technique of using inhaler devices. These interventions will herald a new era of asthma in LMICs with no more asthma deaths and patients living a good quality of life, despite asthma.

## Data Availability

The original contributions presented in the study are included in the article/Supplementary Material, further inquiries can be directed to the corresponding author.
